# Evaluation of Efficiently Removing Secondary Effluent Organic Matters (EfOM) by Al-Based Coagulant for Wastewater Recycling: A Case Study with an Industrial-Scale Food-Processing Wastewater Treatment Plant

**DOI:** 10.3390/membranes13050510

**Published:** 2023-05-12

**Authors:** Yu Cheng, Qiangqiang Cheng, Chengjin Zhao, Xianghao Ren, Yu Wang, Yingying Kou, Kangmin Chon, Myung-Han Ko, Moon-Hyun Hwang

**Affiliations:** 1 Key Laboratory of Urban Stormwater System and Water Environment, Ministry of Education, Beijing University of Civil Engineering and Architecture, Beijing 100044, China; 2 Department of Environmental Engineering, College of Engineering, Kangwon National University, 1 Kangwondaehak-gil, Chuncheon-si 24341, Gangwon-do, Republic of Korea; 3 Department of Integrated Energy and Infra System, Kangwon National University, 1 Kangwondaehak-gil, Chuncheon-si 24341, Gangwon-do, Republic of Korea; 4 ANT21, 34 Gyebaek-ro, Jung-gu, Daejeon 34899, Republic of Korea; 5 Institute of Conversions Science, Korea University, 145 Anam-ro, Sungbuk-gu, Seoul 02841, Republic of Korea

**Keywords:** organic matters, secondary effluent, soluble microbial products, coagulation, humic-like substance, water reuse

## Abstract

The reuse of wastewater has been identified as an important initiative for the sustainable development of the environment; thus, the removal of secondary effluent organic matter (EfOM) to ensure the safety of reused wastewater is the key step and a subject of extensive research. In this study, Al_2_(SO_4_)_3_ and anionic polyacrylamide were selected as coagulant and flocculant, respectively, for the treatment of secondary effluent from a food-processing industry wastewater treatment plant to meet the standard regulatory specifications for water reuse. In this process, the removal efficiencies of chemical oxygen demand (COD), components with UV_254_, and specific ultraviolet absorbance (SUVA) were 44.61%, 25.13%, and 9.13%, respectively, with an associated reduction in chroma and turbidity. The fluorescence intensities (Fmax) of two humic-like components were reduced during coagulation, and microbial humic-like components of EfOM had a better removal efficiency because of a higher Log Km value of 4.12. Fourier transform infrared spectroscopy showed that Al_2_(SO_4_)_3_ could remove the protein fraction of the soluble microbial products (SMP) of EfOM by forming a loose SMP protein complex with enhanced hydrophobicity. Furthermore, flocculation reduced the aromaticity of secondary effluent. The cost of the proposed secondary effluent treatment was 0.034 CNY t^−1^ %COD^−1^. These results demonstrate that the process is efficient and economically viable for EfOM removal to realize food-processing wastewater reuse.

## 1. Introduction

Biological treatment processes are one of the most widely used technologies for wastewater treatment worldwide due to their economic and sustainable characteristics [1]. Although a major fraction of the organic matter in wastewater is degraded during biotreatment, there is a considerable amount of residual organic matter in the secondary effluents identified as secondary effluent organic matter (EfOM). Conventional activated sludge systems (e.g., anaerobic and oxic processes) have not only the insufficient ability to biodegrade the refractory organic compounds (e.g., humic- and fulvic acid-like substances) but also produce soluble microbial products (SMP) which inevitably end up as EfOM and affect the effluent quality [2]. A high concentration of EfOM adversely affects the reuse of wastewater, and the presence of organic matter in the circulating water has become the main factor restricting wastewater reuse [3]. In particular, the presence of organic substances such as humic acid makes the water treatment process more difficult [4]. For the widespread implementation of industrial wastewater treatment and reuse, the government has set strict water reuse standards to force various industries to reduce EfOM concentrations, e.g., when reclaimed water is used as a source of industrial water, the chemical oxygen demand (COD) must be below 60 mg L^−1^ (GB/T 19923-2005, national standard). In addition, in response to the needs of regional development, the application of reclaimed water has been repeatedly written into the construction plan. The recently enacted “Guidance on Promoting Wastewater Resource Utilization (2021)” states that the utilization rate of reclaimed water in the Beijing–Tianjin–Hebei region (China) is specified to increase to over 35% by 2025 [5]. These limits have prompted investigations to eliminate the EfOM from secondary effluents in effective, economical, and engineering-friendly ways to ensure environmental protection and safety of water use.

Coagulation, a physical–chemical process, can neutralize surface electrical charges on particles, reduce electrical double-layer repulsion forces of colloids, and also realize adsorption and complexation with several organic substances [6,7]. Through these mechanisms, many kinds of particulate, colloidal, and dissolved organic matter from EfOM can be removed during coagulation, which is an important opportunity to increase the production capacity of reclaimed water. The EfOM comprises two kinds of organic components, i.e., humic substances and SMPs, depending on their origin [8]. Humic substances in the EfOM range from 17% to 71%, which not only enhance the transportation of heavy metals but also cause harmful byproduct formation during disinfection [1]; SMPs are the major soluble organic matter constituents in EfOM [8]. Coagulation treatment of secondary effluent to remove these two types of organics is effective, and its potential has been proven: Wang et al. [1] concluded that the coagulation process could remove almost half of the humic substances in EfOM; Liu et al. [9] used Al_2_(SO_4_)_3_ and FeCl_3_ as coagulants and obtained SMP removal rates of approximately 25% in both cases. However, the removal efficiency of EfOM is dependent on the composition of secondary effluent, and coagulation treatment of the actual wastewater with complex organic matters needs further confirmation.

Al-based salts have also been widely applied as coagulants and flocculating agents to reduce the EfOM from secondary effluents due to their simplicity, cost-efficiency, and upgradeability. Moreover, Al-based coagulants have good natural organic matter (i.e., humic substances) removal capacity [10], with great potential for EfOM removal, which can produce higher quality water for reuse. Previous studies have reported the characteristics and mechanisms of coagulation using Al-based coagulants. For example, during humic acid removal, it is first adsorbed on the surface of Al(OH)_3(s)_ formed from aluminum hydrolysis, followed by surface complexation between humic acid molecules and surface groups on Al(OH)_3(s)_, under neutral condition [11]. However, secondary effluents have several complex organics. Therefore, it is important to understand the changes in SMPs during coagulation and the changes in the characteristics of humic-like substances with different coagulant dosages. This research should be performed with the actual effluent in an industrial-scale treatment plant for successful practical application.

In this study, an industrial-scale food-processing wastewater treatment plant, which used alum-based coagulation (i.e., Al_2_(SO_4_)_3_) and flocculant (i.e., anionic polyacrylamide; APAM) for the enhancement of secondary effluent quality to meet the local standards for water reuse was investigated to understand the EfOM removal feasibility and cost economy of the coagulation process for treating secondary effluent. The EfOM removal efficiency and change in relative parameters (e.g., turbidity and UV_254_) after coagulation were evaluated. The variation in organic components was monitored based on three-dimensional excitation-emission-matrix (3D-EEM) fluorescence spectra and parallel factor (PARAFAC) analysis after adding different dosages of coagulant. The influence of coagulant dosage on humic-like and SMP-like substances was detected by fluorescence quenching combined with the Ryan–Weber model and the two-dimensional correlation (2D-COS) spectroscopy calculated from synchronous fluorescence (SF) spectra. Structural changes in secondary proteins in the EfOM before and after coagulation were analyzed using Fourier transform infrared (FT-IR) spectroscopy. The effect of Al_2_(SO_4_)_3_ and PAM on the effluent quality after coagulation/flocculation treatment was assessed. Finally, the cost of the treatment was evaluated, and the actual performance of secondary effluent treatment was compared, which provided a basis for promoting the utilization of wastewater resources and improvement of the efficiency and quality of water reuse.

## 2. Material and Methods

### 2.1. Experiment Setup and Operation

In this study, a wastewater treatment plant of a food-processing factory located in Beijing, China, with a stable operation for more than five years, was studied. The mainstream biological units comprise a microaerobic reactor coupled with a membrane bioreactor (MBR). The secondary effluent (Q = 20 m^3^ h^−1^; 150 m^3^ d^−1^) from the MBR then flowed into the coagulation/flocculation reaction section, which was composed of a coagulation reactor (effective volume = 7.1 m^3^; hydraulic retention time (HRT) = 20 min; mixing = 135 rpm × 2.2 kw), a neutralization reactor (effective volume = 7.1 m^3^; HRT = 20 min; mixing = 135 rpm × 2.2 kw), a flocculation reactor (effective volume = 7.1 m^3^; HRT = 20 min; mixing = 60 rpm × 2.2 kw), and a sedimentation tank (effective volume = 155 m^3^). Al_2_(SO_4_)_3_·18 H_2_O and APAM were selected as the coagulant and flocculant with concentrations of 150 g m^−3^ and 1.5 g m^−3^, respectively, which were dissolved completely before adding into the coagulation and flocculation reactor. The addition of NaOH was not required because the pH of secondary effluent was around 7–8 during data collection. The effluent quality met the national standard: “The reuse of urban recycling water-water quality standard for industrial uses” (GB/T 19923-2005).

### 2.2. Jar Test Procedure

Two jar tests were performed in this study. First, the effluent was treated with different dosages of Al_2_(SO_4_)_3_·18 H_2_O (i.e., evenly divided into 10 groups from 0.09 to 0.94 mmol Al L^−1^), and the change in fluorescence characteristics of EfOM was determined. Each sample was filtered through 0.45 μm glass fiber filters (Whatman, Little Chalfont, Buckinghamshire, UK) before analysis. Secondly, the combined effect of Al_2_(SO_4_)_3_ and APAM on the effluent quality after coagulation/flocculation treatment was evaluated. Five different dosages of Al_2_(SO_4_)_3_·18 H_2_O (i.e., 110, 125, 140, 155, and 170 mg L^−1^) were selected based on the variation in the range of the actual addition during the wastewater treatment. The ratio of APAM to Al_2_(SO_4_)_3_·18 H_2_O was 0.012, 0.011, 0.010, 0.009, and 0.008, respectively, for each dosage of coagulant, and these ratios were also determined based on the actual range of addition. All the experimental conditions were consistent with the actual plant operating conditions, except that the settling time for the experiments was 5 min. After settling, the supernatant of each treated group was collected and analyzed.

### 2.3. FT-IR

FT-IR spectra were measured using an FT-IR spectrophotometer (Perkin Elmer Frontier, Waltham, MA, USA) by scanning between wavenumbers of 4000–400 cm^−1^. Each sample was frozen at −33 °C in an ultra-low temperature freezer (Haier, DW-86L338, Qingdao, China) and then dewatered with a freezer dryer at −57 °C and 5 Pa. All FT-IR samples were prepared by pressing a homogenized mixture of 1 mg of freeze-dried sample and 140 mg of potassium bromide (spectrum pure reagent).

### 2.4. Dissolved Organic Matter (DOM) Fluorescence Characteristics Analysis

#### 2.4.1. Three-Dimensional Excitation-Emission-Matrix (3D-EEM) and Parallel Factor Analysis (PARAFAC)

Each sample for dissolved organic matter (DOM) analysis was collected by filtration using 0.45 μm glass fiber filters (Whatman, Little Chalfont, Buckinghamshire, UK). The 3D-EEM spectra of DOM samples were acquired using a fluorescence spectrophotometer (F-7000, Hitachi, Tokyo, Japan). The excitation spectra were scanned every 5 nm from 200 to 450 nm, and the emission spectra were scanned every 5 nm from 200 to 550 nm. Fluorescence regional integration (FRI) was used for calculating the proportion of the five types of fluorescent DOM (FDOM), measured by 3D-EEM. The fluorescence proportion of region *i* (P*_i_*_,n_, %) was calculated according to Equation (1):

(1) $$P_{i,n}\;\left(\%\right)=\frac{\emptyset_{i,n}}{\emptyset_{T,n}}\times 100\%=\frac{{\mathrm{MF}}_{i}\sum_{\mathrm{ex}}\sum_{\mathrm{em}}I\left(\lambda_{\mathrm{ex}}\lambda_{\mathrm{em}}\right){\Delta \lambda }_{\mathrm{ex}}{\Delta \lambda }_{\mathrm{em}}}{\sum_{i=1}^{5}\emptyset_{i,n}}\times 100\%,\;i=I-V$$

where $\emptyset_{i,n}$ is the Ex/Em area referring to the value of region *i*; $\emptyset_{T,n}$ is the Ex/Em area referring to the value of the total region; MF*_i_* is a multiplication factor for each region; I (λ_ex_λ_em_) is the fluorescence intensity for each Ex/Em wavelength pair; Δλ_ex_ and Δλ_em_ are Ex and Em intervals, respectively [12].

PARAFAC was calculated using the DOMFluor toolbox (version 1.7) and N-way toolbox in MATLAB software (MATLAB2021, Natick, MA, USA). The data collected from 44 experimental samples were imported into MATLAB. Modeling was achieved by a series of step-wise procedures performed in a previous study [13], several FDOM components were identified, and the maximum fluorescence intensity (Fmax) was used to characterize the relative concentrations of individual FDOM components [14].

#### 2.4.2. Fluorescence Quenching Titration

The Ryan–Weber model, a commonly used nonlinear fitting model, was used to calculate the coagulation complex constant of FDOM samples and Al-coagulant. This model is mainly based on the following assumptions: (1) the fluorescence quenching of each FDOM component is assumed to be the result of complexation with Al, (2) Al and FDOM have the same and independent binding site or ligand binding, and (3) the stoichiometric ratio of Al to ligand complex is 1:1. The calculation of the R-W model is based on Equation (2):

(2) $$I=I_{0}+\left(I_{ML}-I_{0}\right)(\frac{1}{2K_{M}C_{L}})(1+K_{M}C_{L}+K_{M}C_{M}-\sqrt{{\left(1+K_{M}C_{L}+K_{M}C_{M}\right)}^{2}-4K_{M}{}^{2}C_{L}C_{M}})$$

(3) $$f=\frac{\left(I_{0}-I_{ML}\right)}{I_{0}}$$

where *I*_0_ is the DOM fluorescence intensity of the water sample without Alum; *C_M_* represents the Alum concentration; *I* represents the fluorescence intensity when the Alum concentration is *C_M_*; *C_L_* represents the complexing capacity concentration of Al (mmol L^−1^), and *K_M_* is the Al complexing stability constant. At the same time, *I_ML_* indicates the concentration threshold of Al when the fluorescence intensity stabilizes; f is expressed as the quenching rate, that is, the ratio of the binding group to the total group that was calculated using Equation (3) [15].

#### 2.4.3. Two-Dimensional Synchronous Fluorescence Correlation Spectroscopy (2D-SF-COS)

The synchronous fluorescence (SF) spectra were recorded in the range of 250–550 nm with a constant offset (Δλ = 60 nm) using a fluorescence spectrophotometer (F-7000, Hitachi, Tokyo, Japan) with a scanning speed of 240 nm min^−1^ and a 1 nm scan spacing [15]. The SF spectra with different Al-based coagulant concentrations (see Section 2.2) were used to perform the two-dimensional correlation spectroscopy (2D-COS) using “2D-Shige” software (Kwansei-Gakuin University, Japan).

### 2.5. Analytical Methods

Samples collected from the influent and effluent of the coagulation/flocculation-settling process were analyzed for the different COD fractions, i.e., total COD (TCOD), particulate COD (pCOD; >1.5 μm), colloidal COD (cCOD; 0.45–1.5 μm), and soluble COD (sCOD; <0.45 μm), for which the samples were filtered through 0.45 and 1.5 μm glass-fiber filters [16]. All COD concentrations were measured following the Chinese standard method [17]. The suspended solids (SS) concentration was determined after filtration with 0.45 μm glass-fiber filters. The pH was measured using a portable multimeter (PHB-4, Zsynet, Shanghai, China). The concentration of dissolved organic carbon was quantified using a total organic carbon analyzer (MULTI N/C^®^ 3100, Analytik Jena, Thuringia, Germany). Turbidity was measured using a turbidimeter. UV_254_ was determined by UV absorbance at 254 nm wavelength with a UV–visible–NIR spectrophotometer (LH-3BA, Shenzhen, China). Specific ultraviolet absorbance (SUVA) was calculated using Equation (4) [18]:

(4) $$SUVA\;\left(L\;mg^{-1}m^{-1}\right)=\frac{UV_{254nm}}{DOC}\times 100$$

Chroma is measured as color number (CN) with the absorbance measured at 436 nm (A_436_), 525 nm (A_525_), and 620 nm (A_620_) wavelengths, and calculated using Equation (5) [19]:

(5) $$CN\;\left(cm^{-1}\right)=\frac{A_{436}^{2}+A_{525}^{2}+A_{620}^{2}}{A_{436}+A_{525}+A_{620}}\times 100$$

## 3. Results and Discussion

### 3.1. Performance of the Coagulation Process

The changes in organic matter and wastewater characteristics before and after coagulation are shown in Table 1. The TCOD concentration in the influent was 26.36 mg L^−1^, and that in the effluent was 14.14 mg L^−1^. The average removal efficiency of TCOD was 44.61%. The UV_254_ and SUVA in the influent were 0.263 and 2.25 cm^−1^, which decreased by 25.13% and 9.13% to 0.197 and 2.07 cm^−1^, respectively, in the effluent after coagulation/flocculation treatment.

Compounds that absorb at 254 nm comprise aromatic groups that are often considered biorecalcitrant [20], and the decrease in UV_254_ value after coagulation treatment indicates significantly improved effluent quality. The percentage reduction in UV_254_ absorbing components was lower than that of TCOD, which indicates a more efficient removal of nonaromatic organic matter compared to aromatic constituents [20] after treatment. The lower SUVA value of effluent compared to that of influent indicated its lower aromaticity and the possibility of achieving a lower high-molecule-weight refractory humic-like compounds content through coagulation [8].

The values for the other parameters (i.e., pH, SS, CN, and turbidity) in the influent and effluent are depicted in Table 1. The pH decreased from 7.92 to 7.00, indicating that the process of coagulation/flocculation reduced the pH value of the alkaline influent to neutral. The SS concentration in the influent was 4.56 mg L^−1^, which decreased by 85.01% to 0.66 mg L^−1^ in the effluent after coagulation/flocculation. Because of the efficient removal of SS and TCOD, the CN and turbidity were significantly reduced by 36.65% and 50.35%, respectively, after the coagulation/flocculation process. The turbidity removal efficiency was better than that of TCOD, which was attributed to the difference in coagulation mechanisms of particulate, colloidal, and dissolved organic matter [7]. This suggested that organics contributing to the turbidity were removed efficiently. These phenomena were attributed to the efficient removal of organic matter from secondary effluent.

### 3.2. Change in COD Fractions

**Figure 1:**
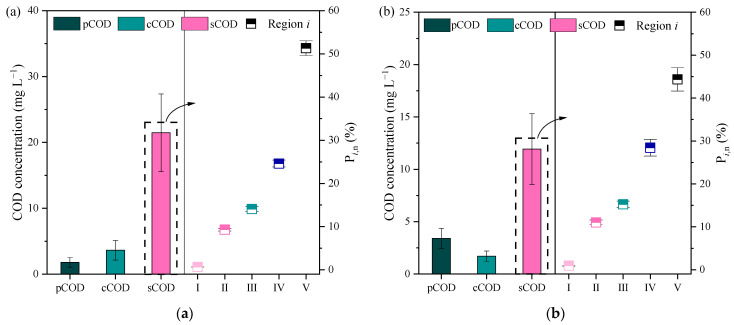
The concentration of COD fractions and organic composition of sCOD (solubleCOD) in the influent (**a**) and effluent (**b**).

Three types of COD fractions (i.e., pCOD, cCOD, and sCOD) were monitored, as shown in Figure 1.

The concentration of pCOD and cCOD in the influent was 1.81 mg L^−1^ and 3.63 mg L^−1^, and those in the effluent were 3.41 mg L^−1^ and 1.71 mg L^−1^, respectively. The contribution of these two types of COD was very less in the TCOD, with an average of 23.06% and 30.03% before and after coagulation. The sCOD decreased from 21.47 mg L^−1^ to 11.93 mg L^−1^, which significantly contributes to TCOD removal. The pH of the influent was approximately 7.9 in this study. According to a previous study report [21], prehydrolyzed aluminum salt solutions may react with the alkalinity present in EfOM, as shown below:

Al_2_(SO_4_)_3_ + 6OH^−^ → 2Al(OH)_3_ + 3SO_4_^2−^

At this pH, the Al hydroxide precipitates, completely dominating all other Al species. Thus, the major coagulation mechanisms for the removal of pCOD and cCOD are adsorption on the precipitates and/or sweep flocculation [22]. The concentration of pCOD in the effluent increased slightly due to the small flocs with adsorbed organic matter remaining suspended in the effluent. The sCOD (i.e., DOM), the major fraction of EfOM, decreased significantly by 44.43%, and changes in its characteristics during treatment were further examined.

In order to understand the changes in DOM components, FRI analysis based on EEM fluorescence spectral data was performed. Five types of DOM components were identified by FRI analysis. Region Ⅰ and Ⅱ contents were identified as protein-like components, constituting 0.67% and 9.31% of DOM in the influent, respectively, showing a slight increase after treatment. Region Ⅲ, a fulvic-like component, changed slightly from 14.08% to 15.23% after coagulation. Region Ⅳ and Ⅴ constituted the highest proportions in the influent and effluent, changed notably with the addition of alum coagulant. Region Ⅳ (i.e., SMP-like component) increased from 24.61% to 28.44%, while Region Ⅴ humic-like substance decreased significantly from 51.32% to 44.35%. These results confirmed that humic-like substances could be efficiently removed by alum [23], and it could contribute to maintaining a low CN in the effluent. Alum-based coagulants have superior natural organic matter (NOM; e.g., humic-like substance) removal capacity based on sweep adsorption and surface complexation [10,11]. However, alum did not seem to have a significant effect on the removal of protein-like components, including SMP. It may be attributed to the fact that SMP-like compounds were more hydrophilic and hence more difficult to remove by Al-based coagulants [24]. To some extent, the significant reduction in humic-like substances caused an increase in the proportions of other substances.

### 3.3. Influence on DOM Components

#### 3.3.1. Component Separation and Dehumification Analysis

**Figure 2:**
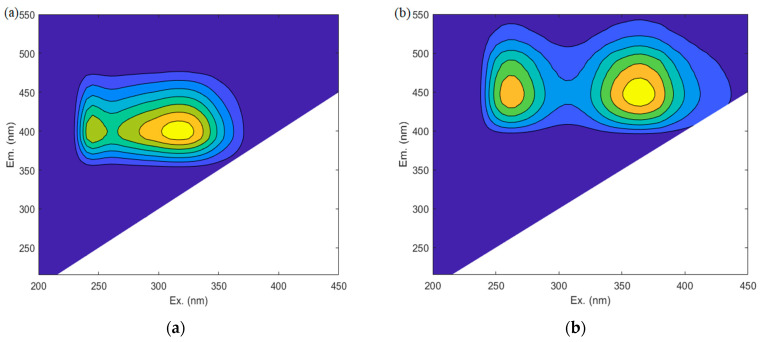

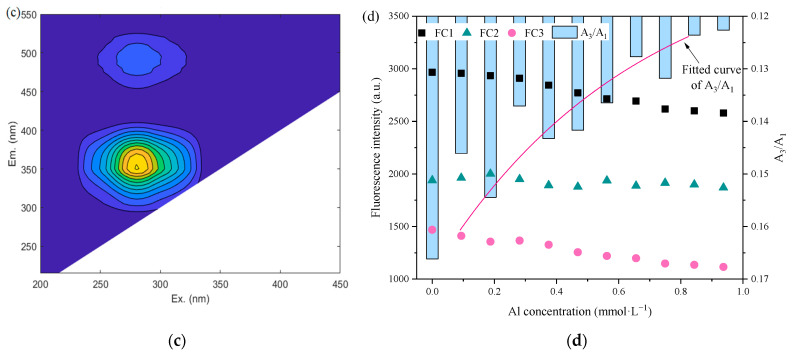
The EEM−PARAFAC characteristics of FC1 (**a**), FC2 (**b**), and FC3 (**c**) of DOM, and (**d**) Fmax and humification degree changes after treatment with different Al concentrations.

Three principal components were identified from the 3D-EEM fluorescence spectra using PARAFAC analysis, and the contour plots of the three components are shown in Figure 2a–c.

The fluorescence component 1 (FC1; Figure 2a), with two peaks at wavelengths λex/λem = 315 (245) nm/400 nm, was assigned to microbial humic-like fluorescence [8]. The fluorescence component 2 (FC2; Figure 2b) with a peak at λex/λem = 365 (260) nm/450 nm was identified as a large-molecular-size humic-like compound [8]. The fluorescence component 3 (FC3; Figure 2c) was a protein-like substance associated with microbial activity and biological productivity that could be regarded as SMP, with a fluorescence peak at 280 nm/250 nm (λex/λem). The analysis indicated that there was a considerable amount of allochthonous organic matter (i.e., humic-like material) in EfOM, and the SMPs were less than the humic-like materials [8]. It was also suggested that these two substances significantly influence EfOM, which was consistent with a report from a previous study [25].

In order to further evaluate the removal efficiency of each component and the effectiveness of dehumification during the addition of alum-based coagulation, fluorescence quenching titration and synchronous fluorescence spectra were established (Figure 2d). There were considerable differences in the changes in DOM components with changes in concentration of Al_2_(SO_4_)_3_. The Fmax of the two humic-like components (i.e., FC1 and FC2) decreased gradually with increasing Al addition. The Fmax of FC1 was much higher than that of FC2, which indicated that microbial humic-like substances are major constituents of EfOM in this study. Unlike the variations in FC1 and FC2, the change in Fmax of FC3 was inconsistent at different dosages of coagulant, which meant the removal efficiency of SMP could not be accurately estimated by fluorescence intensity using EEM spectra. The value of A3 (400–550 nm)/A1 (300–350 nm) was positively correlated with humification degree [26], and it decreased from 0.17 to 0.12 when Al concentration increased from 0.00 to 0.94 mmol L^−1^. This indicated that the humific acid content of effluent gradually decreased when FC1 and FC2 were efficiently removed from EfOM.

The two types of hydrophobic organic components (i.e., FC1 and FC2) can interact with Al_2_(SO_4_)_3_ through complexation with hydroxyl ions on the surface [7,8]. The Log Km and f values of FC1 and FC2 are shown in Table 2; according to the Ryan–Weber model fitting, the different removal characteristics between FC1 and FC2 were examined. The different contributions of the two types of humic-like substances on the dehumification of the secondary effluents during the process of coagulation were evaluated. For FC1, the values of Log Km and f were 4.12 and 0.12, respectively; those for FC2 were 4.10 and 0.22. It was suggested that FC1 was more sensitive to Al-based coagulant, compared to FC2, either from the aspect of complex constants or from the degree of quenching. Hence, it can be confirmed that microbial humic-like substances can be efficiently removed by Al_2_(SO_4_)_3_ addition, and large-molecular-size humic-like compounds were also complex during this process, although their removal efficiency was not as effective as that of the former.

#### 3.3.2. Variation in SMP

**Figure 3:**
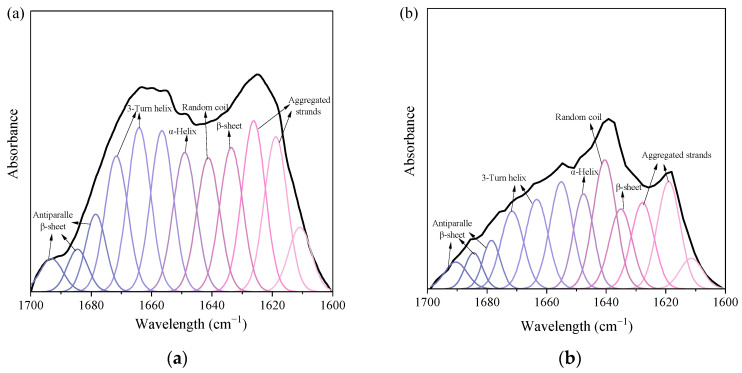
FTIR spectra and Second derivative resolution enhanced and curve−fitted amide I region (1700−1600 cm^−1^) for proteins of the samples: (**a**) influent; (**b**) effluent.

The effect of Al_2_(SO_4_)_3_ on the forms and contents of the corresponding functional groups (i.e., C=O and N-H) of SMP-like substances, and the change in the composition ratio of the related secondary structures, were examined by FTIR analysis. FTIR spectra at wavelength range 1700–1600 cm^−1^ (i.e., amide Ⅰ region) are shown in Figure 3.

Several types, i.e., aggregated strands, β-sheet, random coil, α-helix, 3-turn helix, and antiparallel β-sheet/aggregated strands, were sorted from secondary structures of SMP protein [27]. Results confirmed that adding Al_2_(SO_4_)_3_ reduced the total areas of peaks, and the cutback in the total areas of peaks indicated an effective reduction in protein content (Table 3). The proportions of α-helix and random coil increased from 10.39% and 10.43% to 12.24% and 15.77%, respectively, while that of β-Sheet decreased from 10.43% to 8.95%. The ratio of α-helix to (β-sheet + random coil) was 0.498 in the influent, and in the effluent, it was 0.495. A high percentage of α-helix and a low percentage of β-sheet could lead to the tight structure of the protein, while the low value of the ratio of α-helix and (β-sheet + random coil) confirmed that the loose structure of protein molecule promoted more hydrophobicity in SMP protein [27]. Hence, coagulation with Al_2_(SO_4_)_3_ could reduce the proteins in SMP from EfOM, aided by a loose SMP protein structure and enhanced hydrophobicity of SMP proteins.

#### 3.3.3. Characteristics of DOM Removal

**Figure 4:**
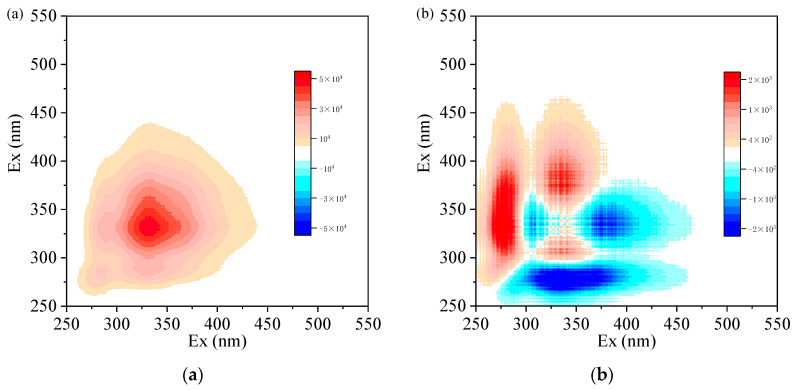
Two−dimensional correlation maps generated based on SFS of DOM samples treated with different Al concentrations: (**a**) 250−550 cm^−1^ synchronous spectra; (**b**) 250−550 cm^−1^ asynchronous spectra. Red represents positive correlations, and blue represents negative correlations; higher color intensity indicates a stronger positive or negative correlation.

To understand the changes in DOM with different dosages of Al_2_(SO_4_)_3_ during coagulation, synchronous and asynchronous maps were generated from SF spectra. Three major auto-peaks were revealed in the synchronous map (Figure 4a). The change in band intensity followed this order: 330 > 362 > 280 nm. The bands at 330 nm and 362 nm were related to humic-like fluorescence compounds, and the former band may be derived from microbial processes as microbial humic-like components [15,28]. This analysis showed that the DOM changed with time in the following order: humic-like > protein-like substances and microbial humic-like components have an advantage over other humic-like substances in the process of coagulation. This result was consistent with the EEM analysis of dehumification that the removal of microbial humic-like substance plays a major role in this process. Furthermore, the cross-peak of the two bands showed a positive correlation, implying that the three component signals consistently changed during the process of coagulation with Al_2_(SO_4_)_3_.

The asynchronous map displays distinctive characteristics compared with the synchronous map, which reveals the degree of the sequential changes at the three different wavelengths with the addition of Al_2_(SO_4_)_3_ (i.e., the extent to which the fluorescence intensity changes at one wavelength either leads or lags behind that at the other wavelength) [28], shown in Figure 4b. According to Noda’s rule [29], the changes in the bands with time followed this order: 330 > 362 > 280 nm. These results imply that microbial humic-like fractions might be removed earlier than the other fractions, and the protein-like substances displayed a weaker capturing capacity by Al-based coagulant (i.e., Al_2_(SO_4_)_3_). Hence, humic-like components of EfOM could have more chances of getting removed by Al_2_(SO_4_)_3_ than other fractions during coagulation.

### 3.4. Optimization of Coagulation/Flocculation Efficiency

**Figure 5:**
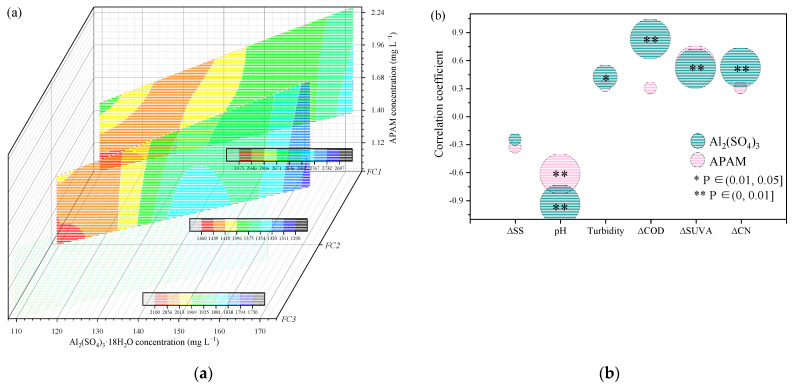
The influence of coagulant and flocculant concentration on FC1, FC2, and FC3 components of DOM (**a**) and effluent pollutant indices (**b**).

The concentration of Al_2_(SO_4_)_3_·18 H_2_O and APAM influence the efficiency of EfOM removal during coagulation and flocculation processes. The change in EEM-PARAFAC characteristics of FC1, FC2, and FC3 of FDOM in EfOM treated by different concentrations of Al_2_(SO_4_)_3_·18 H_2_O and APAM is exhibited in Figure 5a. The three components had blue shifts in different degrees due to the gradually increased concentration of Al_2_(SO_4_)_3_·18 H_2_O and APAM, which positively influenced FDOM removal. With the change in concentration of these two treatment chemicals, based on the range of concentration applied in this wastewater treatment plant for EfOM removal, the three components of FDOM exhibited different removal characteristics. When the concentration of coagulant was around 110–120 mg L^−1^, the Fmax of FC1, FC2, and FC3 decreased notably with the increase in the ratio of flocculant to coagulant concentration. The flocculant concentration would influence the efficiency of microbial humic-like substance removal in this situation. However, the influence of a higher proportion of flocculant on three components removal was not always positive. Its efficiency stabilized when the dosage of flocculant exceeded a certain amount (i.e., the ratio of APAM to Al_2_(SO_4_)_3_·18 H_2_O was 0.009 in this study). When the concentration of coagulant increased, the Fmax of humic-like substances decreased significantly compared to protein-like substances; humic-like components located at the high band of Ex/Em, in particular, displayed a reduction rate of Fmax up to 14.10%, in response to increased concentrations of coagulant combined with an equal proportion of the flocculant. However, it has been reported that a higher concentration of PAM might form large amounts of loose-structure flocs that negatively affect organics removal [30]. Hence, the removal efficiencies of the three components did not continue to increase with the increase in the concentration of Al_2_(SO_4_)_3_·18 H_2_O; and did not change significantly when the concentration of Al_2_(SO_4_)_3_·18 H_2_O exceeded 155 mg L^−1^.

The correlation between the concentration of Al_2_(SO_4_)_3_·18 H_2_O and APAM and the efficiency of coagulation is shown in Figure 5b to demonstrate the effect of coagulant and flocculant treatment on pollutant removal. The removal rate of SS was not proportional to the concentration of coagulant and flocculant, and it may be attributed to the effects of MBR treatment which results in very less SS in secondary effluent. The turbidity was significantly negatively correlated (*p* < 0.05) with the concentration of coagulant while the removal rate of COD, SUVA, and CN was significantly positively correlated (*p* < 0.01) with the coagulant concentration. It indicated coagulation by Al_2_(SO_4_)_3_ resulted in a major reduction in EfOM from a secondary effluent and efficiently guaranteed the effluent quality of WWTP. APAM, as a flocculant, balances the pH and reduces SUVA contents. These results also suggested that the concentrations of coagulant and flocculant were positively related to the effluent quality in the range of dosage applied in the study, and the adjustment of chemical dosages can effectively address the changes in secondary effluent quality (i.e., variation in EfOM).

### 3.5. Integrated System Economic Evaluation

The cost of building or upgrading treatment systems is one of the most critical barriers to the widespread utilization of industrial wastewater reuse [31]. The total cost for applying a coagulation/flocculation and sedimentation integrated treatment system for the secondary effluent treatment of the industrial-scale food-processing wastewater treatment plant was estimated. Table 4 summarizes the resources used and the cost calculation.

The construction cost (manufacturing of treatment tanks and mechanical cost) was estimated to be 4,000,000 CNY, and the electrical power cost was estimated at 0.98 CNY t^−1^. On the other hand, the chemical costs were estimated to be about 0.45 CNY t^−1^ for the coagulant and 0.08 CNY t^−1^ for the flocculant. The maintenance cost was considered to be 5000 CNY a^−1^. The average cost of secondary effluent treatment from this study was 1.51 CNY t^−1^ with a 44.61% COD removal rate, which implies a cost of 0.034 CNY t^−1^ %COD^−1^; Su et al. [2] had applied Fenton coupled with biological aerated filter to treat EfOM, and this treatment cost was 0.045 CNY t^−1^ %COD^−1^ to meet the effluent standard, which was higher than that estimated in this study. Moreover, it is also more economical compared with ozonation (estimated cost: 0.03–0.07 CNY t^−1^ gCOD^−1^) to improve effluent quality [32]. Compared to other processes [33], Al-based coagulant has the advantages of low cost and high efficiency and has great practical application potential in water reuse for industrial uses. Overall, Al_2_(SO_4_)_3_ and APAM-based coagulation/flocculation is a comparatively economic treatment process for efficient EfOM removal to realize food-processing wastewater reuse.

## 4. Conclusions

In this study, Al_2_(SO_4_)_3_ and APAM were chosen as coagulant and flocculant, respectively, for the treatment of secondary effluent from an industrial-scale food-processing wastewater treatment plant to meet the regulatory standards for wastewater reuse. The EfOM was removed efficiently with a 44.61% COD removal rate during this process, and both dearomatization and dehumification were achieved to different degrees. Coagulation played a key role in the removal of humic-like components of EfOM, especially the organics from microbial humic-like components. The degree of removal of protein-like components of SMP was lower than humic-like components; however, coagulation can produce a loose SMP protein structure and enhance the hydrophobicity of SMP proteins. Flocculation can further reduce the aromaticity of the secondary effluent and strengthen the sedimentation. The cost of secondary effluent treatment in this study was 0.034 CNY t^−1^ %COD^−1^, which showed that Al-based coagulant had a viable application value for EfOM removal to reach the goal of food-processing wastewater reuse.

## Figures and Tables

**Table 1:** The performance of coagulation/flocculation process under treating secondary effluent.

Parameter	Influent	Effluent	Removal Efficiency (%)
TCOD (mg L^−1^)	26.36 ± 7.20	14.41 ± 3.57	44.61 ± 6.53
UVA_254_ (cm^−1^)	0.263 ± 0.002	0.197 ± 0.005	25.13 ± 1.64
SUVA (L mg^−1^ m^−1^)	2.25 ± 0.03	2.07 ± 0.03	9.13 ± 1.35
CN (cm^−1^)	0.020 ± 0.001	0.012 ± 0.002	36.65 ± 9.49
SS (mg L^−1^)	4.56 ± 1.50	0.66 ± 0.58	85.01 ± 12.00
Turbidity (NTU)	1.19 ± 0.28	0.63 ± 0.45	50.35 ± 34.43
pH	7.92 ± 0.28	7.00 ± 0.05	11.52 ± 1.25

TCOD, total COD; UVA_254_, ultraviolet absorbance at 254 nm; SUVA, specific ultraviolet absorbance; CN, color number; SS, suspended solids.

**Table 2:** Complexing stability constant of Al with DOM by the Ryan–Weber model.

Component	Log Km	f	R^2^
FC1	4.12	0.12	0.9197
FC2	4.10	0.22	0.7289

**Table 3:** Band assignments for the protein secondary structures of dissolved organic matter.

Wavelength cm^−1^	Secondary Structures	Influent	Effluent
		Proportion of Area (%)	Proportion of Area (%)
1616–1626	Aggregated strands	20.86	18.04
1629–1640	β-sheet	10.43	8.95
1640–1645	Random coil	10.43	15.77
1649–1650	α-Helix	10.39	12.24
1655–1669	3-Turn helix	20.78	17.20
1676–1694	Antiparallel β-sheet/aggregated strands α-Helix/(β-sheet +random coil)	14.21	12.80

**Table 4:** Total capital investment for coagulation process of secondary effluent treatment.

Parameter	Value
Q	150 m^3^ d^−1^
No. of working days per year	365 d
tw	7 h d^−1^
Construction cost (designing & treatment units manufacturing & mechanical cost)	CNY 4,000,000
Electrical costs	CNY 0.98 t^−1^
Chemical costs Al_2_(SO_4_)_3_·18 H_2_O	CNY 0.45 t^−1^
APAM	CNY 0.08 t^−1^
Maintenance cost	CNY 5000 a^−1^

## Data Availability

The data presented in this study are available on request from the corresponding author.
